# Exosomes: A Rising Star in Failing Hearts

**DOI:** 10.3389/fphys.2017.00494

**Published:** 2017-07-13

**Authors:** Jun-Yan Xu, Gui-Hao Chen, Yue-Jin Yang

**Affiliations:** State Key Laboratory of Cardiovascular Disease, Department of Cardiology, Fuwai Hospital, National Center for Cardiovascular Diseases, Chinese Academy of Medical Science and Peking Union Medical College Beijing, China

**Keywords:** exosomes, cardiac dysfunction, microRNAs, proteins, heat shock proteins, stem cells

## Abstract

Although exosomes were previously recognized as a mechanism for discharging useless cellular components, growing evidence has elucidated their roles in conveying information between cells. They contribute to cell–cell communication by carrying nucleic acids, proteins and lipids that can, in turn, regulate behavior of the target cells. Recent research suggested that exosomes extensively participate in progression of diverse cardiovascular diseases (CVDs), such as myocardial infarction, cardiomyopathy, pulmonary arterial hypertension and others. Here, we summarize effects of exosome-derived molecules (mainly microRNAs and proteins) on cardiac function, to examine their potential applications as biomarkers or therapeutics in CVDs.

## Introduction

Cardiovascular diseases (CVDs) are the leading cause of mortality worldwide. According to reports from the World Health Organization, over 17.5 million people died from CVDs in 2012, accounting for 31.4% of all deaths (WHO, 2014). CVDs can appear rapidly, such as in acute myocardial infarction (AMI), or progress slowly over years, as with chronic heart failure. Although percutaneous coronary intervention, coronary artery bypass grafting and medications can significantly preserve cardiac function after heart attack, pathological cardiac remodeling that has already occurred cannot be reversed and may lead eventually to heart failure.

Exosomes are nano-sized (30–150 nm) extracellular vesicles released from almost all types of cells (Kowal et al., 2016) and widely existing in body fluids, including plasma, urine, saliva, pleural effusions, pericardial effusions and cerebrospinal fluid (Bard et al., 2004; Pisitkun et al., 2004; Michael et al., 2010; Street et al., 2012; Kuosmanen et al., 2015). Previous studies demonstrated that exosomes contain various bioactive molecules, such as nucleic acids (DNA and RNA), proteins and lipids and, thus, can be involved in proximal and distal intercellular communication. So far, exosomes have been shown to influence immune modulation (Bianco et al., 2007), tumor invasion (Cai et al., 2012), regeneration, and degenerative processes (Ma et al., 2017), under both physiological and pathological conditions. Recently, exosomes were shown to participate in regulating cardiac function in health and disease, by delivering signaling molecules. Such research not only increased understanding of mechanisms of CVDs but also suggested potential applications of exosomes in treating or predicting CVDs. Currently, stem cell therapy has limitations and produces unsatisfactory results. Exosomes may serve as alternatives, based on their favorable properties, including stability, biocompatibility, biological barrier permeability, low toxicity and low immunogenicity (Xitong and Xiaorong, 2016). This review provides an overview of the biology of exosomes, their regulatory roles in failing hearts and future perspectives on their potential therapeutic applications.

## Exosomes

It is widely recognized that cells secrete two main types of extracellular vesicles, exosomes and microparticles/shedding microvesicles. These vesicles have various sizes and are generated by different mechanisms. Exosomes, relatively smaller in size (30–150 nm), are generated by the traditional endosomal pathway. Microparticles/shedding microvesicles (100–1,000 nm) are generated by the direct outward budding of cell membranes. Also, other types of extracellular vesicles, identified according to their sizes and surface markers, include apoptotic bodies (500–2,000 nm), and large oncosomes (1,000–10,000 nm) (Minciacchi et al., 2015). Among these vesicles, exosomes are the focus of this review.

### Exosome biogenesis and release

**Figure 1:**
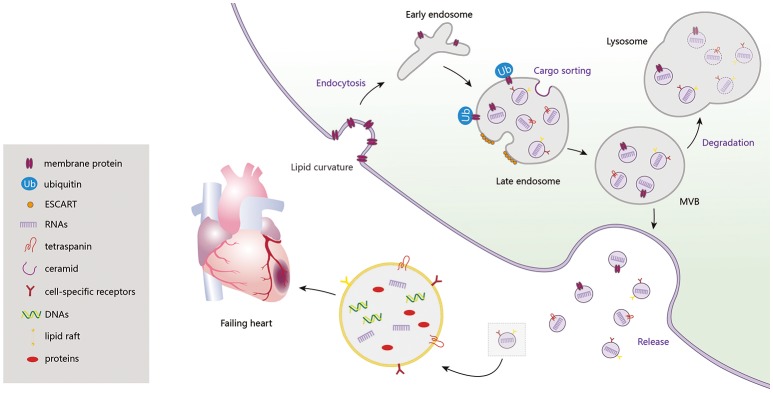
Exosome biogenesis, release and degradation. Exosomes contain a large amount of DNAs, RNAs, proteins (CD9, CD63, CD81, Alix, TSG101, Hsps, RAS-related proteins, CXCR4, TNF-α, Shh, HSF-1, Clusterin, and etc.) and lipids, which regulating the cardiac function in failing heart.

Exosome biogenesis is induced, at different stages, by multiple mechanisms. Among them, the endosomal sorting complex required for transport (ESCRT) machinery, containing ESCRT-0, I, II, III and accessory proteins, is the best known. Lipid bilayer curvature enables endocytosis of membrane proteins and formation of inward budding vesicles (early endosomes). During endosome maturation, ESCRT-0 recruits ubiquitinated proteins and ESCRT-I, II, and III induce budding of intraluminal vesicles (ILVs), along with sorting of the eventual exosome cargoes (Cocucci and Meldolesi, 2015). ESCRT-III sequentially facilitates fission of ILVs in the lumens of late endosomes, forming multivesicular bodies (MVBs) (Chiaruttini et al., 2015). In addition to the ESCRT machinery, formation of sphingolipid ceramides on the endosomal membrane was shown to induce coalescence of small microdomains into larger domains, promoting domain-induced budding (Trajkovic et al., 2008). Another pathway of exosome formation is tetraspanin mediated organization of amyloidogenic protein premelanosome proteins, participating in cargo partitioning into ILVs in an ESCRT-independent manner (van Niel et al., 2011). When these MVBs fuse with the plasma membrane, ILVs are discharged as exosomes from cells, acting on adjacent or remote cells through autocrine or paracrine processes. Otherwise, MVBs are degraded, with participation of autophagosomes, and their contents are recycled by fusion with the intracellular lysosomes (Figure 1).

### Exosome interactions

**Figure 2:**
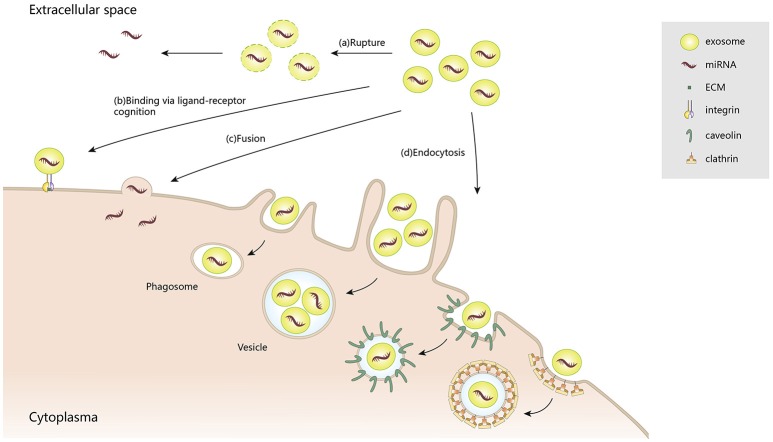
The multiple patterns of exosome-induced intracellular communication.

After being secreted into the extracellular space, exosomes communicate with target cells via diverse mechanisms: (1) releasing effective components into the extracellular space; (2) binding to the cell surfaces; (3) fusion with plasma membranes; and (4) endocytosis by target cells. Although exosomes are relatively stable compartments, under certain conditions they may liberate their components, such as interleukin-1β (Qu et al., 2007) and vascular endothelial growth factor receptor 2 (VEGFR2) (Jarad et al., 2017). Also, those exosomes that maintain structural integrity can selectively bind to the cell surface via ligand–receptor recognition. For example, tumor-derived exosomes are delivered to different organs, depending on their various encased integrins, through binding to extracellular matrix (ECM) on the cell surface (Hoshino et al., 2015). Such directional delivery enables them to release their contents, through plasma membrane fusion, into specific recipient cells. Notably, a high lipid content in exosomes and acidic microenvironments can facilitate their fusion with cell membranes (Maas et al., 2017). In addition to membrane fusion, exosomes can also be internalized by endocytosis, for example, clathrin-mediated endocytosis, macropinocytosis, phagocytosis, and caveolae-mediated endocytosis. As confirmed by high throughput microscopy, exosomes derived from rat pheochromocytoma (PC12) cells were endocytosed into bone marrow-derived mesenchymal stromal cells (BMMSCs) through clathrin-mediated endocytosis and macropinocytosis (Tian et al., 2014). In phagocytic cells, exosomes were internalized more efficiently, via phagocytosis, than in non-phagocytic cells (Feng et al., 2010). Caveolae, which is typical flask-like shape (50–100 nm) plasma membrane, may also contribute to exosome endocytosis (Pols and Klumperman, 2009; Nanbo et al., 2013). Because of their extensive participation in intercellular transportation and transmission of signaling cargoes, exosomes are recognized as key contributors to pathological and physiological processes (Figure 2).

### Molecular components of exosomes

Exosomes can often carry specific cargos of DNA, RNA, proteins and lipids, distinct from those found in their parent cells. Single-stranded DNA, double-stranded DNA, mitochondrial DNA and oncogene amplifications, such as c-myc, were identified in exosomes (Guescini et al., 2010; Balaj et al., 2011; Thakur et al., 2014). Studies suggested that a circulating exosomal DNA mutation was a pancreatic biomarker in patients, enabling cancer prediction and assessment (Kahlert et al., 2014; Yang et al., 2017). Recently, inhibition of exosome secretion was shown to lead to nuclear DNA accumulation in the cytoplasm, potentially activating innate immune responses and causing reactive oxygen species dependent DNA damage. The latter effect can induce senescence-like cell cycle arrest or cellular apoptosis. This study demonstrated that exosome secretion maintained cellular homeostasis by removing harmful cytoplasmic DNA from cells (Takahashi et al., 2017). However, information about exosomal DNA is limited and more research is needed to clarify whether it is functional or only represents contaminants from dead cells. Exosomal microRNA (miRNA) is an important biological cargo in exosomes, participating extensively in cellular crosstalk. It is notable that miRNAs are not randomly incorporated into exosomes. Instead, specific miRNAs are selectively exported to exosomes, at constant exosome:miRNA ratios. In addition, specific exosomal miRNA content can clearly change under various pathophysiological conditions (Zhang et al., 2015). According to previous research, there are four underlying modes for miRNA sorting, through pathways dependent on: (1) neural sphingomyelinase 2 (Kosaka et al., 2013); (2) miRNA motif and sumoylated heterogeneous nuclear ribonucleoproteins (Villarroya-Beltri et al., 2013); (3) the 3′-end of miRNA (Koppers-Lalic et al., 2014); and (4) miRNA induced silencing complex (Frank et al., 2010). In addition to nucleic acids, exosomes are universally known to shuttle proteins. Among these, some, including CD9, CD63, CD81, TSG101, and Alix, are involved in exosome generation, release and uptake (Sahoo and Losordo, 2014) and theoretically are present in all types of exosomes. As a consequence, these proteins are usually called exosomal markers and are commonly used to characterize exosomes. More specific proteins, capable of exerting biological effects by activating corresponding pathways in their recipient cells, are also present in exosomes (Mackie et al., 2012; Feng et al., 2014a; Kang et al., 2015; Pironti et al., 2015). In addition, exosomes were reported to be rich in lipids, such as glycosphingolipids, sphingomyelin, cholesterol and phosphatidylserine, compared with their parent cells (Llorente et al., 2013). However, the biological functions of exosomal lipids in physiological or pathophysiological states are largely unknown.

### Exosome isolation

Exosomes can be isolated from either body fluids, such as plasma, urine, saliva, nasal secretions and milk, or supernatants prepared from cells into which they were released (Witwer et al., 2013). So far, five types of methods are used to isolate exosomes, based on their unique specific characteristics, such as density, size, shape and marker proteins. These methods were developed using differential ultracentrifugation, ultrafiltration, precipitation, immunoaffinity or microfluidics. Isolation principles and protocols of these techniques were previously described in detail (Li et al., 2017). Differential ultracentrifugation is currently the most widely used method for separating exosomes, with a recent study reporting that most (about 81%) of the members in the International Society for Extracellular Vesicles isolate exosomes for their research by ultracentrifugation, irrespective of the starting materials (Gardiner et al., 2016). However, all existing techniques have advantages and disadvantages and there is no universal isolation method (Li et al., 2017). Exosomes used in various studies may have been contaminated by proteins, lipoproteins, polymeric materials and other vesicles, like microvesicles. Therefore, these contaminants should be removed, to obtain exosomes that are as pure as possible, before exploring their biological effects. For example, exosomes isolated by precipitation are often contaminated by proteins (Lobb et al., 2015) and polymeric materials (Li et al., 2017). Methods to remove such contaminants include combining precipitation with immunoaffinity capture. Moreover, parameters of the techniques used for isolation can affect the biological features of the resulting vesicles, complicating experimental results. For instance, two previous studies proposed conflicting mechanisms underlying the procoagulant properties of microvesicles from patients with sickle cell anemia (Shet et al., 2003; van Beers et al., 2009). Authors of both studies claimed that the procoagulant characteristics were attributable to “microparticles,” but the vesicles acquired by the two research groups were not equivalent. The two studies utilized dramatically different isolation protocols, producing microvesicles and exosomes in one study and microvesicles in the other. A major difference was ultracentrifugation of vesicles at 18,890 × g (van Beers et al., 2009) and 100,000 × g (Shet et al., 2003) in the two studies. As a consequence, it is necessary for investigators to thoroughly understand the isolation methods and choose the most appropriate strategies for isolating exosomes in their studies.

Despite numerous improvements over the past few years, isolation of exosomes remains technically challenging. To provide necessary material for increasing numbers of exosome research studies and to facilitate application of exosomes into clinical practice, more efforts are needed to develop an isolation method with high yields, efficiency, purity and throughput and, if possible, decreased costs.

In this review, if not otherwise indicated, the exosomes were isolated by ultracentrifugation. Table 1, presenting stem-cell derived exosomes, and Table 2, describing exosome experiments related to proteins, both list the isolation methods to give a better description.

**Table 1:** The biological effects of stem cell-derived exosomal miRNAs.

**Cell types**	**Tissues**	**Isolation methods**	**Pretreatment/modifications**	**MiRNAs**	**Pathological status**	**Biological effects**	**References**
MSCs	SD rats bone marrow	Ultracentrifugation (3,000 rpm 30′ → 13,000 rpm 30′ → 0.2 μm filter → 36,000 rpm 3 h)	None	miR-223	LPS-induced heart injury	Pro-inflammatory cytokine↓	Wang X. et al., 2015
		Precipitation(SBI)	Ischemic preconditioning	miR-22	MI	Cardiomyocyte apoptosis↓Ventricular fibrosis↓	Feng et al., 2014b
		Precipitation (SBI)	GATA-4 overexpression	miR-19a	MI	Cardiomyocyte apoptosis↓ Cardiomyocte hypoxia resistance↑	Yu et al., 2015
	Human endometrium	Precipitation (SBI)	None	miR-21	MI	Angiogenesis↑ Cardiomyocyte survival↑	Wang et al., 2017
CPCs	Human right atrial appendage	Ultracentrifugation (3,000 g 15′ → 0.2 μm filter → 100,000 g 90′)	None	miR-210	MI	Cardiomyocyte apoptosis↓	Barile et al., 2014
				miR-132	MI	Angiogenesis↑	
	SD rat heart	Ultracentrifugation (10,000 g 35′ → 100,000 g 70′ → 100,000 g 70′ twice)	12h hypoxic conditioning	miR-15b, miR-17, miR-20a, miR-103, miR-199a, miR-210, and miR-292	I/R injury	Angiogenesis↑ Ventricular fibrosis↓	Gray et al., 2015
Sca-1+ CPCs	C57BL/6 mouse heart	3,000 rpm 15′ → 0.22 μm precipitation (SBI)	H_2_O_2_	miR-21	Hypoxia	Cardiomyocyte apoptosis↓	Xiao et al., 2016
CDCs	Human heart	Precipitation (SBI)	None	Unknown	MI	Angiogenesis↑ Cardiomyocyte hypertrophy↓ Ventricular fibrosis↓	Gallet et al., 2017
	Human interventricular septum	Precipitation (SBI)	None	miR-146a and other miRNAs	MI	Angiogenesis↑ Cardiomyocyte proliferation↑ Cardiomyocyte survival↑	Ibrahim et al., 2014
	Human and WKY rat heart	Ultrafiltration → Centrifugation (no detailed information given)	None	miRNA-181b	I/R injury	Cardiac function↑ Infarct size↓ Macrophage distinctive polarization↑	de Couto et al., 2017
ESCs	C57BL/6 mouse heart	800 g 5′ → 14,000 g 20′ → 100,000 g 1 h on 30% sucrose-D_2_O solution	None	miR-290 family (miR-291, miR-294 and miR-295)	MI	Angiogenesis↑ Cardiomyocyte proliferation↑ Cardiomyocyte survival↑	Khan et al., 2015
iPSCs	C57BL/6 mouse heart	Ultracentrifugation (1,000 rpm 10′ → 0.22 μm filter → precipitation (poly-ethylene glycol) → 3,000 rpm 30′)	H_2_O_2_	miR-21, miR-210	I/R injury	Cardiomyocyte apoptosis↓	Wang Y. et al., 2015

**Table 2:** Exosomal proteins in the failing heart.

**Proteins**	**Sources of exosomes**	**Isolation methods**	**Stimuli/Modifications**	**Experimental models**	**Biological effects**	**References**
**Hsp**						
Hsp60	Cardiomyocyte	Ultracentrifugation with sucrose (100,000 g 75′) or Precipitation (SBI kit)	ethanol	*In vitro*	Reflects the extent of ethanol-induced injury (probably)	Malik et al., 2013
Hsp20	Cardiomyocyte	Precipitation (SBI kit)	Hsp20 overexpression	*In vitro*	HUVEC tube formation↑	Zhang et al., 2012
				*In vivo*	Myocardial angiogenesis↑	
		Ultracentrifugation (3,000 rpm 20′ → 13,000 rpm 30′ → 0.22 μm filter → 120,000 g 2 h)		*In vitro* (hyperglycemia)	Cardiomyocyte death↓ EC death↓	Wang et al., 2016
				*In vivo* (streptozocin)	Cardiac apoptosis↓ Cardiac fibrosis↓ Cardiac hypertrophy↓ Microvascular rarefaction↓	
Hsp70	Plasma	Ultracentrifugation (1,600 g 20′ → 10,000 g 30′ → 100,000 g 60′ twice)	None	*In vitro* (hypoxia/reoxygenation)	Cardiomyocyte death↓	Vicencio et al., 2015
				*In vivo* (I/R)	Myocardial infarct size↓	
Peptides from Hsp70	Polymersome (synthetic nanovesicles)	Unknown	Functionalization with peptides from Hsp70	*In vitro* (hypoxia/reoxygenation)	Cardiomyocyte death↓	Radenkovic et al., 2016
**OTHER PROTEINS**						
HSF-1	Sca-1+ stem cell	Precipitation (SBI kit)	Heat shock	*In vitro* (oxygen glucose deprivation)	Sca-1+ stem cell death↓	Feng et al., 2014a
				*In vivo* (AMI)	Cardiomyocyte apoptosis↓ Cardiac function↑	
Clusterin	Pericardial Fluid	Ultracentrifugation (1,200 g 20′ → 10,000 g 30′ → 0.45 μm filter → 0.22 μm filter → 100,000 g 1 h)	None	*In vivo* (AMI)	Cardiac function↑	Foglio et al., 2015
Shh	CD34+ cell	Ultracentrifugation (400 g 15′ → 14,000 g 30′ → 100,000 g 60′ with sucrose → 100,000 g 60′)	Shh overexpression	*In vivo* (AMI)	Myocardial infarct size↓ Cardiac function↑	Mackie et al., 2012
CXCR4	MSC	Precipitation (SBI kit)	CXCR4 overexpression	*In vitro* (hypoxia)	EC tube formation↑ Cardiomyocyte apoptosis↓	Kang et al., 2015
				*In vivo*	Cardiac function↑	
AT_1_R	Cardiomyocyte	Ultracentrifugation (1,000 g 15′ → 12,000 g 20′ → 20,000 g 20′ → 0.22 μm filter → 100,000 g 70′)	Osmotic stretch; cardiac pressure overload	*In vitro*	AT_1_R-rich exosome release↑	Pironti et al., 2015
				*In vivo* (cardiac pressure overload)	Cardiac function↓	
Unknown	Fibroblast	Ultracentrifugation (500 g 10′ → 20,000 g 20′ → 100,000 *g* 70′ with sucrose → 100,000 g 70′)	Ang II	*In vitro*	Cardiomyocyte hypertrophy↑	Lyu et al., 2015
				*In vivo*	Cardiac hypertrophy↑	
TNF-α	Cardiomyocyte	Ultracentrifugation (300 g 10′ → 16,500 g 20′ → 0.22 μm filter → 100,000 g 2 h → 100,000 g 70′)	Hypoxia	*In vitro*	Ventricular remodeling (probably)↑	Yu et al., 2012

### Exosome characterization

Observing and characterizing exosomes is very challenging because of their small sizes, below detection limits of many common techniques. For example, flow cytometers, conventionally used to characterize cells, cannot be used to analyze unmodified exosomes because the vesicles are too small to be distinguished from noise. However, exosomes can be detected by flow cytometry when tagged with fluorophores (van der Pol et al., 2012), or stained with fluorophore-bound antibodies after binding to latex beads (Ostrowski et al., 2010). The most straightforward method to characterize exosomes is electron microscopy (EM). Under EM, exosomes are visible as double-membrane particles, ranging in diameter from 30 to 150 nm. However, although many studies concluded that exosomes were “cup-shaped” vesicles (Lehmann et al., 2008; Palanisamy et al., 2010; Twu et al., 2013; Vicencio et al., 2015), this shape was shown to be an artifact of sample drying for EM (Thery et al., 2009). As exosomes are generated by the endosomal pathway, certain proteins, including CD9, CD63, CD81, Alix, and TSG101, associated with this pathway are present in exosomes and used for their identification (Lotvall et al., 2014). Western blotting is the most common technique for semi-quantification of these marker proteins and to confirm the presence of exosomes. Other methods for identifying exosomes, such as nanoparticle tracking analysis (Gardiner et al., 2013) and dynamic light scattering (Sahoo et al., 2011) are based on light scattering and Brownian motion of particles. These two methods are reliable and are often used for semi-quantification of exosomes. Nevertheless, it is important to note that the particles identified by these techniques are not only exosomes. Particles of similar sizes, like cellular debris or lipoproteins, are also detectable by these two methods. Besides these commonly used methods, previous studies also described other techniques for characterizing exosomes, such as atomic force microscopy (Sharma et al., 2010), micronuclear magnetic resonance, small-angle X-ray scattering (van der Pol et al., 2013) and resistive pulse sensing (van der Pol et al., 2014). When characterizing exosomes, many studies combine two or more isolation methods, like EM, western blotting and nanoparticle tracking analysis, in the same study (Vicencio et al., 2015; Blans et al., 2017; Helwa et al., 2017).

## Exosomal miRNAs

miRNAs, once known as small temporal RNAs (Lee et al., 1993; Reinhart et al., 2000), are highly conserved non-coding RNAs with only 20–25 nucleotides. Because of their specific roles in post-transcriptional regulation of gene expression, a growing number of studies demonstrated that they were important in both physiological and pathological conditions (Lau et al., 2001; Lai, 2002; Rana, 2007). Prior studies confirmed miRNA alterations with progression of various CVDs, thus they can serve as novel potential biomarkers. Recently, increasing evidence suggested that exosomes can transfer miRNAs between cells through internalization of exosomes (Vickers and Remaley, 2012). Indeed, exosomal miRNAs participate in variety of basic cellular functions, including cell proliferation, apoptosis, cytokine production, immune modulation and metastasis, via paracrine or endocrine mechanisms (Huang-Doran et al., 2017). Hence, we propose that these intriguing exosome-derived miRNAs are significant in regulation of cardiac function.

### Exosomal miRNAs in pathological cardiac remodeling

Cardiac dysfunction is considered to be the ultimate result of the adaptive and maladaptive cardiac remodeling that leads to abnormal alterations in cardiomyocyte biology, myocardium and ventricular geometry (Mann and Bristow, 2005). Regarding the characteristics of exosome-derived miRNAs, it is significant to consider how they promote or suppress this process. This part of the review summarizes relevant literature over recent decades, focusing on three topics, cardiomyocyte hypertrophy, impaired myocardial angiogenesis and ECM degradation (Cohn et al., 2000).

#### Cardiomyocyte hypertrophy

Cardiac hypertrophy is one of the major changes in cardiac structure contributing to adaptation to cardiac overload. During cardiac hypertrophy, various kinds of cells deliver hypertrophic signals to cardiomyocytes via exosomal miRNAs.

In a mouse model of myocardial infarction (MI), exosomal miRNA-133a levels were significantly decreased in infarcted and peri-infarcted myocardium, accompanied by cardiomyocyte death (Kuwabara et al., 2011). Through exosomal transportation, miRNA-133a may be captured by cells adjacent to non-infarcted areas, exerting inhibitory effects on hypertrophy (Care et al., 2007). miRNA-21_3p (miRNA-21^*^) is selectively packaged into exosomes from cardiac fibroblasts and endocytosed by cardiomyocytes, rather than being simply attached to the cell surface. Cardiomyocyte size measurements confirmed that miRNA-21^*^ transported by exosomes was crucial for mediating cellular hypertrophy *in vitro*. This effect was attenuated by pharmacological inhibition of miRNA-21^*^ in mice with angiotensin II (Ang II)-induced cardiac hypertrophy (Bang et al., 2014). After pretreatment of cardiac fibroblasts with Ang II, exosomes derived from these cells induced increased expression of renin, angiotensinogen, Ang II receptor types 1 (AT_1_R) and Ang II receptor types 2 (AT_2_R), while downregulating angiotensin converting enzyme 2, in cardiomyocytes. Hence, more Ang II was produced and released by cardiomyocytes, which could activate the upregulated AT_1_R and AT_2_R on the same cells, leading to hypertrophy. It was suggested that these effects were induced by cardiac fibroblast-derived exosomal proteins and miRNAs, including miRNA-132 (Lyu et al., 2015). Additionally, adipose tissue is a crucial organ present extensively in the human body and was implicated in hypertrophic signal transmission. A recent study demonstrated that PPAR-γ activation in adipocytes increased secretion of miRNA-200-contained exosomes, producing cardiomyocyte hypertrophy through the mammalian target of rapamycin (mTOR) pathway (Fang et al., 2016).

#### Impaired myocardial angiogenesis

Aberrant angiogenesis in the heart is a pathological condition involved in impaired angio-adaptation, decreased capillary angiogenesis, myocyte–capillary mismatch and myocardial micro-arteriopathy. Aberrant angiogenesis is believed to accelerate the transition from adaptive to pathological cardiac remodeling or heart failure (Wanner et al., 2016). Exosomal miRNAs were shown by several studies to be responsible for regulating tumor-associated vessel formation (Skog et al., 2008; Grange et al., 2011; Umezu et al., 2013). However, the exact mechanism by which exosomal miRNAs affect angiogenesis in cardiac dysfunction remains unknown.

Exosomal miRNA-146a, induced by a 16 kDa prolactin fragment, increased apoptosis and decreased proliferation of endothelial cells (ECs), effects detrimental to the cardiac microvasculature in peripartum cardiomyopathy (PPCM) (Halkein et al., 2013). Conversely, miRNA-146a in cardiosphere-derived cell (CDC) exosomes (isolated by precipitation) stimulated myocardial angiogenesis and enhanced cardiac function in mice with MI. Thus, exosomal miRNA-146a may participate in sophisticated communication mechanisms, under different pathological conditions (Ibrahim et al., 2014). Another study reported that glucose starvation for 48 h stimulated cardiomyocytes to secrete exosomes that could promote tube formation by enhancing proliferation of human umbilical venous endothelial cells (HUVECs). This effect was attributed to the angiogenic miRNAs (miRNA-17, 19a, 19b, 30c, and 126) in the exosomes (Garcia et al., 2015). Because acute heart failure is linked to a locally pathologic microenvironment, it is likely that decreased glucose uptake and oxidation in cardiomyocytes would increase biogenesis of exosomes containing miRNA, leading to compensatory angiogenesis in the myocardium (Zhabyeyev et al., 2013). On the contrary, excessive glucose may impede normal angiogenesis in the heart. In adult Goto-Kakizaki mice (a model for type 2 diabetes), cardiomyocytes inhibited cardiac EC migration, proliferation and tube formation through exosomal transfer of miRNA-320. These findings provided clues about the mechanisms of cardiac impairment in diabetes mellitus patients (Wang et al., 2014).

#### ECM degradation

ECM and its regulators have comprehensive functions in the heart and vasculature. ECM remodeling occurs in areas of cell apoptosis or necrosis, as a result of imbalances in ECM synthesis, maturation and degradation. The major histological alteration in heart failure is myocardial fibrosis, primarily caused by progressive collagen deposition in the myocardium (Bouzeghrane et al., 2005; Braunwald, 2013). miRNA-29 family members, including miRNA-29a, miRNA-29b1/b2, and miRNA-29c, are important regulators in heart, liver, lung and kidney fibrosis, affecting expression of collagens, integrins and matrix metalloproteinases (MMPs) (van Rooij et al., 2008; Cushing et al., 2011; Qin et al., 2011; Roderburg et al., 2011). In a mouse model of type 2 diabetes, an exercising group had notably increased levels of cardiomyocyte-derived exosomes (isolated by ultrafiltration) in the extracellular space, vessel walls and regions adjacent to cardiomyocytes and had upregulated miRNA-29b and miRNA-455. Through decreased release of MMP-9, one determinant of ECM degradation, exosomes enriched in miRNA-29b and miRNA-455 attenuated fibrosis and cardiomyocyte uncoupling (Chaturvedi et al., 2015).

### Exosomal miRNAs in CVDs

#### MI

MI, as a sequela of ischemic heart diseases/coronary artery diseases (CAD), is the leading cause of sudden death or chronic heart failure in the world. Despite improved revascularization strategies, the number of patients undergoing heart failure is still increasing. Hence, it is necessary to identify high-risk patients as early as possible and to mitigate subsequent ischemia/reperfusion (I/R) injury.

So far, troponin T, troponin I, creatine kinase and myoglobin have been extensively used to detect early stages of acute coronary syndrome (ACS) and, to some degree, to predict prognosis of patients with ACS. However, miRNAs are emerging as potential candidates because of their specificity, stability and wide distribution in body fluids. Plasma miRNA-208b and miRNA-499 levels were significantly elevated after AMI, with corresponding alterations in levels of troponin T and creatine phosphokinase, markers for cardiac injury (Corsten et al., 2010). However, another clinical study reported no changes in miRNA-208b (not detected in plasma) or miRNA-499 (not shown) in AMI patients. These investigators observed significant upregulation (at least 8-fold, compared with the control group) of miRNA-1, miRNA-133a, miRNA-133b, and miRNA-499-5p and downregulation of miRNA-122 and miRNA-375 (D'Alessandra et al., 2010). In accordance with this study, serum levels of miRNA-1 and miRNA-133a were also significantly increased in patients with unstable angina pectoris and AMI prior to troponin T elevation, making them promising biomarkers for predicting ACS. Indeed, studies clarified that these miRNAs were released into the circulation, along with cardiomyocyte exosomes, by calcium stimulation (Kuwabara et al., 2011). Moreover, exosomal miRNA was identified as a prognostic factor in AMI patients. In serum obtained from patients, at a median of 18 d after AMI onset, exosomal (isolated by precipitation) miRNA-192, miRNA-194 and miRNA-34a, all p53-responsive miRNAs, were increased significantly in those patients who developed ischemic heart failure within 1 year (Matsumoto et al., 2013). Because of its protection from RNase degradation by sequestration in exosomes and formation of protein–miRNA complexes, miRNA can be stabilized in serum, urine and even saliva (Michael et al., 2010). Urine samples were analyzed and compared with blood samples to monitor exosomal miRNA levels, temporally and spatially, in AMI mice. Urine exosomal miRNA-1 levels were increased over 50-fold at 24 h after AMI, but the elevation was delayed compared with in blood samples, consistent with the observations in AMI patients (Cheng et al., 2012). Though obtaining urine samples is relatively non-invasive, we do not believe that indirect analysis of urine exosomal miRNAs has obvious advantages at present, because miRNA excretion may be affected by differences in rates of urine formation and kidney metabolism.

Previous studies showed that exosomes could serve as mediators for amelioration of cardiac I/R injury. Repeated remote ischemic conditioning (5 cycles of 5 min bilateral hindlimb ischemia and 5 min reperfusion, once per d for 4 wk) improved both systolic and diastolic function of the left ventricle, despite comparable MI sizes in experimental and control groups. Exosomal miRNA-29a levels in the marginal area of infarction were increased in accordance with those in serum in the remote ischemic conditioning group. Furthermore, C2C12 cells (skeletal myoblasts) were stimulated by hypoxia to release exosomes rich in miRNA-29a. Taken together, these findings were consistent with observations that exosomes were released into the blood circulation by hindlimb skeletal muscle cells and then endocytosed into cardiomyocytes in mice subjected to remote ischemic preconditioning (Yamaguchi et al., 2015). When delivered to the ischemic/infarcted myocardium from remote ischemic organs or from the heart itself, via exosomes, miRNAs can regulate autophagy, providing a protective response against apoptosis (Maiuri et al., 2007; Giricz et al., 2014). Hypoxia induced miRNA-30a upregulation and enrichment into cardiomyocyte exosomes (isolated by precipitation) and, also, decreased typical intracytoplasmic autophagic vacuoles. Inhibition of miRNA-30a or exosome release attenuated cardiomyocyte apoptosis by maintaining autophagy (Yang et al., 2016). On the contrary, by increasing miRNA-144 expression in circulating exosomes (isolated by precipitation), remote ischemic preconditioning suppressed the autophagy negative regulator p-mTOR, thus reducing infarct size and improving cardiac function (Li et al., 2014).

#### Cardiomyopathy

Cardiomyopathies describe a heterogeneous group of myocardial diseases, leading to cardiovascular death or disabilities related to progressive heart failure. Cardiomyopathies are generally divided into four subgroups, hypertrophic, dilated, restrictive, and arrhythmogenic cardiomyopathy, with diverse etiologies (Maron et al., 2006). PPCM manifests as dilated cardiomyopathy (DCM), but the miRNA alterations in PPCM and DCM are not exactly the same. Consistent with results in PPCM-like mice, exosomal miRNA-146a levels were increased in both serum and left ventricular tissue in PPCM patients, whereas exosomal miRNA-146a remained unchanged in DCM patients. In patients receiving standard anti-heart failure therapy and bromocriptine for about 6–8 wk, blood sample analysis (mean followup, 5 ± 2.5 months) showed significantly decreased exosomal miRNA-146a levels, compared with patients in the acute phase, with concomitantly improved cardiac function. Hence, exosomal miRNA-146a may act as a specific biomarker to identify patients with peripartum heart failure (Halkein et al., 2013). Diabetic cardiomyopathy is one of the most severe complications of diabetes mellitus, accompanied by impaired left ventricular systolic or diastolic function, ventricular hypertrophy, interstitial fibrosis and myocardial microvascular rarefaction (Jia et al., 2016). Exosomes (isolated by precipitation) released from diabetic cardiomyocytes are believed to adversely affect proliferation and migration of ECs by transferring miRNA-320. Such effects were impeded either by adding GW-4869, an exosome inhibitor, to cardiomyocytes, or by transfection with an inhibitory rno-miRNA-320 adenovirus (Wang et al., 2014). More studies are needed to illuminate the roles of exosomal miRNAs as mediators in cardiomyopathies.

#### Pulmonary arterial hypertension (PAH)

PAH is an abnormal state in the pulmonary vasculature, characterized by increased pulmonary arterial pressure and eventually leading to right-sided heart failure (Galie et al., 2009). The pathogenesis of PAH is generally recognized as including sustained vasoconstriction and progressive, detrimental vascular remodeling, caused by endothelial dysfunction and aberrant proliferation of fibroblasts and smooth muscle cells (Tuder et al., 2009).

Exosome-mediated transport, via miRNA molecules, is critical for modulating cell–cell communication in PAH. When subjected to PAH stimuli, pulmonary artery smooth muscle cells (PASMCs) exhibited increased levels of miRNA-143-3p, leading to increased cell migration. Through exosome transfer, miRNA-143-3p can also increase migration and proliferation of pulmonary arterial endothelial cells (PAECs). Furthermore, a consistent upregulation of miRNA-143-3p levels in the lung and right ventricle was observed in mouse models for PAH. In addition, qPCR studies detected miR-143-3p in both cardiomyocytes and cardiac fibroblast cells. These results indicated that PASMC-derived exosomes also interact with cardiomyocytes and fibroblasts in the heart, via miRNA-143-3p, facilitating progression of PAH-induced right-sided heart failure (Deng et al., 2015). Mesenchymal stromal cell (MSC)-derived exosomes (MEX) deliver therapeutic signals to PASMCs and PAECs. On one hand, MEX inhibited PASMC proliferation in response to serum-derived mitogens. On the other hand, MEX also totally abrogated activation of hypoxia-induced transducer and activator of transcription 3 (STAT3), a key mediator leading to pulmonary hypertension, in human PAECs. In a mouse model of hypoxic pulmonary hypertension, MEX inhibited macrophage influx and suppressed release of pro-inflammatory/pro-proliferative factors, helping to suppress inflammation and ameliorate pulmonary hypertension, right ventricular hypertrophy and vascular remodeling. These benefits were attributed to exosomal miRNA-16, miRNA-21, and let-7b pre-miRNA (Lee et al., 2012).

#### Valvular heart disease (VHD)

VHD is a structural or functional abnormality of cardiac valves, leading, if untreated, to progressive cardiac dysfunction. The etiologies of VHD are very complex, including heritable factors, inflammation, endocardial disorders, myocardial diseases, neoplasm, degeneration, iatrogenic factors, drugs and physical agents and infiltration; there is, as well, an idiopathic form (Boudoulas et al., 2013). Exosomal miRNAs influence cardiac valve formation and, thus, may participate in pathogenesis of congenital VHD. During the process of cardiac valve formation, hyaluronan, regulated by hyaluronan synthase, was identified as a key component of cardiac jelly (primary cardiac valve) in the atrioventricular canal (Lagendijk et al., 2013). miRNA-23 suppressed hyaluronan synthase, preventing excessive deposition of hyaluronan in cardiac jelly. Aberrant alterations in miRNA-23 levels led to destruction of hyaluronan homeostasis and, consequently, valvular defects in newborn hearts (Lagendijk et al., 2011). With mathematical modeling, these investigators demonstrated that miRNA-23 was transferred by EC-derived exosomes (Lagendijk et al., 2013). However, this study focused on only congenital VHD. More research is needed to elucidate correlations between exosomal miRNA and other forms of VHD, considering the complexity of their etiologies.

### Potential therapeutics: stem cell-derived exosomal miRNAs

In both pre-clinical and clinical studies, stem cell therapy was proposed as beneficial for cardiac function in MI and ischemic heart failure (Amado et al., 2005; Schuleri et al., 2009; Bartunek et al., 2013). To date, various kinds of stem cells, including MSCs, cardiac progenitor cells (CPCs), embryonic stem cells (ESCs), and induced pluripotent stem cells (iPSCs), delivered using multiple approaches, were studied to identify the optimal strategy against maladaptive cardiac remodeling and decreased cardiac function (Freyman et al., 2006). However, controversies remain regarding immunogenicity, safety (tumorigenesis) and efficacy (low survival rate and low retention rate) of stem cell therapies. Recent studies demonstrated that stem cells could sort bioactive substances into exosomes and, thus, influence the heart in a paracrine manner. Considering the problems associated with stem cell therapy, stem cell-derived exosomes are promising potential alternatives for treating CVDs (Table 1).

#### MSC-derived exosomes

MSCs, also known as mesenchymal or multipotent stromal cells, have long been the most widely studied stem cell type for treating CVDs. MSCs are obtained from multiple tissues, including bone marrow, skeletal muscle, adipose tissue, umbilical cord, synovium, circulatory system, dental pulp, amniotic fluid, fetal blood, liver, and lung (Phinney and Prockop, 2007). Based on growing evidence, MSC enhances cardiac regeneration in a paracrine manner. In immunodeficient mice with AMI, despite improved cardiac function resulting from intravenous administration of human MSCs, no apparent engraftment of human MSCs was observed in the heart at 3 wk after AMI. In fact, human MSCs were shown to exert their effects on the infarcted heart via secretion (paracrine) rather than differentiation mechanisms (Iso et al., 2007). Many studies confirmed that exosomes released by MSC are major factors influencing the efficacy of MSC transplantation (Lai et al., 2010). In addition, exosomes delivered intravenously had no adverse effects on liver and renal function and caused no hemolysis or vascular or muscle stimulation, no systemic anaphylaxis, no pyrogenic reaction and no hematological changes. Such evidence supported the safety of MSC exosomes for pre-clinical and clinical trials (Sun et al., 2016).

MSC-derived exosomes derived from various tissues and exposed to different pretreatments or modifications can regulate cardiac function differently. Multiple organ failure, such as cardiac and renal dysfunction, which may ultimately lead to death, is very common in patients with sepsis (Takasu et al., 2013). BMMSC-derived exosomes suppressed LPS-induced pro-inflammatory cytokine release from macrophages and protected cardiomyocytes against LPS associated injury in a sepsis model, induced by cecal ligation and puncture. These beneficial effects were attributed to miRNA-223 transfer (Wang X. et al., 2015). After ischemic preconditioning, BMMSC-derived exosomes (isolated by precipitation) had higher miRNA-22 levels, compared with those not preconditioned, and suppressed cardiomyocyte apoptosis and subsequent left ventricular fibrosis in mice with MI. These benefits were attributed to a mechanism targeting methyl CpG binding protein 2 (Feng et al., 2014b). Gene modification of BMMSC, to overexpress GATA-4, increased miRNA-19a levels in exosomes (Exo^GATA−4^). In a hypoxic environment, exosomal (isolated by precipitation) miRNA-19a enhanced resistance of the cardiomyocytes to hypoxia and decreased cardiomyocyte apoptosis. *In vivo*, direct intramyocardial injection of Exo^GATA−4^ in an AMI mouse model led to improved cardiac function and decreased infarct areas 4 wk later. Indeed, miRNA-19a containing Exo^GATA−4^ regulated cardiac function by inhibiting phosphatase and tensin homolog, thereby activating the Akt and ERK signaling pathways (Yu et al., 2015). Interestingly, in a recent study, pretreatment of cardiac stem cells (CSCs) with BMMSC-derived exosomes (isolated by precipitation) decreased survival rate and angiogenic activity of the CSCs after transplantation, potentially because of regulation of miRNAs (Zhang et al., 2016). Compared with BMMSC and adipose-derived MSCs, human endometrial MSCs had more cytoprotective and other potentially therapeutic effects when co-cultured with neonatal cardiomyocytes and HUVECs or when delivered to rats with MI. Upregulation of exosomal (isolated by precipitation) miRNA-21, along with activation of the downstream Akt pathway, contributed to cell survival, angiogenesis and subsequent recovery of cardiac function (Wang et al., 2017).

#### CPC-derived exosomes

CPCs are present in the hearts of several species, including mouse, rat, dog, pig, and human. Different types of CPCs are identified based on their properties and surface markers, including side population, c-kit+, Sca-1+, Islet 1+, SSEA-1+, and CDCs (Bollini et al., 2011). Compared with other types of stem cells, CPCs have tissue-specific patterns and are pre-committed to cardiovascular lineages, which may give them unique regeneration potential in CVDs. The CADUCEUS clinical trial showed that intracoronary administration of autologous CDCs decreased scar size, increased viable myocardium and improved regional function in infarcted myocardium (Makkar et al., 2012; Malliaras et al., 2014). Recently, in a post-infarct heart failure model, CPC-derived exosomes enhanced recovery of cardiac function, supporting the biological activities attributed to these extracellular vesicles (Kervadec et al., 2016).

With its high exosome content, CPC-conditioned medium can inhibit HL-1 cardiomyocyte apoptosis and enhance tube formation by HUVECs *in vitro*. Based on analysis of miRNAs, exosomes in CPC-conditioned medium are enriched in miRNA-210 and miRNA-132. Further experiments revealed that these CPC-derived exosomes can repress ephrin A3 and PTP1b, via miRNA-210, thereby reducing cellular apoptosis. In addition, such exosomes can enhance tube formation by downregulating the target of miR-132, RasGAP-p120. In an *in vivo* study, intramyocardial injection of CPC-derived exosomes improved left ventricular ejection fraction in mice with MI (Barile et al., 2014). Another study showed that hypoxia (12 h) induced CPCs to load exosomes with anti-fibrotic and pro- angiogenetic miRNAs, including miRNA-15b, miRNA-17, miRNA-20a, miRNA-103, miRNA-199a, miRNA-210, and miRNA-29. When such hypoxic CPC-derived exosomes were applied to ECs and TGF-β-stimulated fibroblasts, they enhanced tube formation and decreased pro-fibrotic gene expression, respectively. Furthermore, these exosomes improved cardiac function and decreased fibrosis after I/R injury (Gray et al., 2015). In addition, when CPCs (Sca-1+) were pretreated with H_2_O_2_, which mimics oxidative stress in the heart after ischemic diseases, they released exosomes (isolated by precipitation) rich in miRNA-21. These exosomal miRNA-21 had anti-apoptotic effects on H9c2 cardiomyocytes under oxidative stress, increasing programmed cell death 4 and suppressing cleaved caspase-3 (Xiao et al., 2016). In pig models of AMI, intramyocardial delivery of CDC-derived exosomes (isolated by precipitation) decreased the no-reflow area and infarct size and maintained left ventricle ejection fraction, whereas intracoronary delivery had no effect. Additionally, intramyocardial delivery of CDC-derived exosomes in convalescent MI led to decreased left ventricle collagen content, decreased cardiomyocyte hypertrophy and increased vessel density, along with decreased scar size and preservation of left ventricle ejection fraction (Gallet et al., 2017). Analysis of miRNAs indicated that enrichment of miRNA-146a, in particular, in CDC-derived exosomes (isolated by precipitation) may be responsible for their benefits in infarcted hearts. However, as administration of miRNA-146a mimics simulates only part of the salutary effect of CDC-derived exosomes, there must be other miRNAs or other kinds of substances participating in cardiac regeneration (Ibrahim et al., 2014). Recently, studies showed that CDC-derived exosomal (isolated by ultrafiltration) miRNA-181b preserved cardiac function after I/R injury by targeting protein kinase C δ in macrophages, inducing a distinctive polarization state (de Couto et al., 2017).

#### ESC- and iPSC-derived exosomes

Pluripotent stem cells, including ESCs and iPSCs, have great potential for cardiac regeneration, because of their unparalleled differentiation ability, under appropriate stimulation (Shimoji et al., 2010). However, because of concerns about immunogenicity, teratogenic effects, and ethical issues, clinical use of ESCs is currently limited (Grinnemo et al., 2006; Blum and Benvenisty, 2008). In addition, ESCs and iPSCs have similar problems regarding cell retention and survival when transplanted to the heart. Recent research demonstrated, however, that exosomes derived from ESCs or iPSCs had analogous cardioprotective effects by delivering miRNA molecules to target cells, making them excellent candidates for promoting cardiac regeneration.

In mice with MI, intramyocardial delivery of ESC-derived exosomes significantly increased left ventricular contractility and function, as a result of improved survival and proliferation of cardiomyocytes and increased myocardial neo-vascularization. *In vitro* and *in vivo* studies consistently demonstrated that ESC-derived exosomes promoted CPC survival and proliferation, suggesting that these vesicles interacted with CPCs and activated their endogenous repair mechanisms. Indeed, elevated levels (at least 2.8-fold) of ESC-specific miRNA-290 family members, including miRNA-291, miRNA-294 and miRNA-295, were detected in CPC after pretreatment with ESC-derived exosomes. In addition, miRNA-294 mimics stimulated the effects of ESC-derived exosomes on CPCs, along with increasing Akt phosphorylation, pluripotency regulator protein LIN28 levels and mRNA expression of *c-myc* and *Klf4* in CPCs (Khan et al., 2015). Under H_2_O_2_-induced oxidative stress, iPSC-derived exosomes had anti-apoptotic effects on H9c2 cardiomyocytes, concomitant with decreased caspase-3/7 activation. In a mouse model of myocardial I/R injury, myocardium preconditioned with iPSC-derived exosomes displayed less cardiomyocyte apoptosis at 24 h after reperfusion, an effect attributed to exosomal miRNA-21 and miRNA-210 transfer (Wang Y. et al., 2015).

## Exosomal proteins

Exosomes are also enriched in proteins, in addition to miRNAs. These proteins exist either on the surface or in the lumens of exosomes. Generally, they can be divided into two categories, common and specific. Common proteins, like Alix and TSG101 (Gourlay et al., 2017), are detectable in exosomes released from almost all cell types, under all circumstances, because they are involved in exosome biogenesis. However, the specific proteins are loaded into exosomes only by cells in certain physiological or pathophysiological states (Malik et al., 2013; Foglio et al., 2015; Pironti et al., 2015). For example, clusterin was detected in exosomes derived from pericardial fluid from patients with AMI, but not in that from normal subjects (Foglio et al., 2015). Both the common and specific proteins can affect target cells. Exosomal proteins influence target cells in three ways, by functioning as new receptors on the cell surface (Pironti et al., 2015), directly binding to receptors already existing on the cell surface (Vicencio et al., 2015) or being released by exosomes into target cells (Feng et al., 2014a). Some exosomal proteins are beneficial to cells, while others may be ineffective or detrimental. Thus, on one hand, there is interest in loading exosomes with therapeutic agents and applying them to clinical practice. On the other hand, there is a need to prevent release or even production of exosomes rich in harmful components. Dozens of studies reported that functional proteins can be loaded into exosomes and thus affect target cells (Mackie et al., 2012; Kang et al., 2015; Radenkovic et al., 2016), supporting the promise of producing exosomes with specific therapeutic effects. In addition, exosomal proteins can serve as biomarkers for diagnosing diseases or predicting prognosis of patients, because exosome cargoes can reflect the physiological or pathophysiological states of the parent cells (Table 2).

### Heat shock proteins (Hsps)

Hsps are a family of proteins with key functions in protein trafficking, protein folding, and cell signaling (Azad et al., 2015). Hsps are induced by stresses, such as high temperature, and they are important for maintaining normal cellular functions during a pathophysiological state. With progression of diseases like heart failure and cardiac hypertrophy, the levels of certain specific Hsps increase. These were shown to serve as chaperones or co-chaperones, for example, to prevent protein misfolding or refold denatured proteins (Benjamin and McMillan, 1998). Many studies showed that Hsps could be transferred by exosomes to both neighboring and distant cells. Exosomes carrying specific types of Hsps were shown to affect cardiac function.

#### Hsp70

Hsp70 was identified in numerous studies as a protective factor in heart failure (Naka et al., 2014) and myocardial I/R injury (Radford et al., 1996; Okubo et al., 2001). Recently, plasma exosomes, which carry Hsp70 on their surfaces, were shown to attenuate myocardial reperfusion injury in rats. In *in vitro* studies, exosomes derived from plasma, containing Hsp70, bound to toll-like receptors 4 (TLR4s) on cardiomyocytes and activated the ERK1/2-p38MAPK-Hsp27 signaling pathway (Vicencio et al., 2015). This exciting result supported the therapeutic use of exosomes for reperfusion injury. However, this outcome cannot be directly applied to clinical practice because, to protect the heart from I/R injury, the volume of plasma required to isolate enough exosomes would be approximately 25% of the total plasma volume of a healthy rat. Based on their previous research, and taking this shortcoming into consideration, the same investigators developed a method of producing synthetic exosomes-polymersomes, functionalized with either KSTGKANKITITNDKGRLSK or TKDNNLLGRFELSG peptides from Hsp70 (Radenkovic et al., 2016). Under conditions of simulated I/R injury, the protective effects of these novel nanovesicles on cardiomyocytes were confirmed *in vitro*. Large-scale production of such synthetic exosomes makes them promising as a therapeutic strategy for alleviating I/R injury. Another strategy to translate the therapeutic effects of exosomal Hsp70 into clinical practice would be to specifically stimulate cells to secret exosomes loaded with Hsp70 and, thus, increase plasma levels of these vesicles. It was shown that preconditioning fibroblasts with isoflurane, but not hypoxia, increased levels of Hsp70-loaded exosomes in the conditioned medium (Kraemer et al., 2015). Further studies are needed to demonstrate whether these increased exosomes would be cardioprotective.

#### Hsp20

It was reported that circulating Hsp20 was increased in transgenic (TG) mice with cardiac-specific Hsp20 overexpression, compared with in wildtype mice. In addition, capillary density was significantly enhanced in Hsp20-overexpressing hearts, compared with in non-TG hearts. An *in vitro* study showed that myocytes overexpressing Hsp20 secreted more Hsp20 through a novel mechanism, involving exosomes (isolated by precipitation) produced under stress. Furthermore, extracellular Hsp20 was shown to promote angiogenesis by interacting with the VEGFR2 on ECs and activating downstream signaling involving Akt and ERK (Zhang et al., 2012). Cardiomyocytes in type 2 diabetic rats mediated anti-angiogenesis by transferring miRNA-320, via exosomes, to neighboring ECs. Exosomal miRNA-320 downregulated expression of proangiogenic proteins like Hsp20, insulin-like growth factor (IGF-1) and Ets2 (a transcription factor required for EC survival) (Wang et al., 2014). Specific overexpression of Hsp20 in cardiomyocytes in the TG mice significantly alleviated streptozocin-induced hypertrophy, apoptosis, fibrosis and blood vessel rarefaction in the heart and thus improved cardiac function. These effects were largely attributed to transfer of high levels of exosomal Hsp20, p-Akt, survivin and superoxide dismutase from Hsp20-TG cardiomyocytes to other cardiac cells (Wang et al., 2016). Therefore, exosomal Hsp20 is potentially useful for alleviating diabetes-induced injuries and thus attenuating diabetes associated cardiac dysfunction.

#### Hsp60

Hsp60 is a mitochondrial and cytosolic protein. In heart failure, Hsp60 is translocated to the plasma membrane and released into the plasma (Lin et al., 2007). Hsp60 levels were reportedly doubled in end-stage heart failure (Knowlton et al., 1998). Previous studies (Gupta and Knowlton, 2007; Malik et al., 2013) demonstrated that Hsp60 was released by cardiomyocytes via exosomal pathway and that levels of exosomal Hsp60 were substantially increased by exposure of the cardiomyocytes to ethanol. First, it was reported that, by activating the TLR4-MyD88-IRAK-1 pathway, Hsp60 promoted inflammation in the heart and induced cardiomyocyte apoptosis, facilitating progression of heart failure (Li et al., 2011). Second, chronic alcohol-consumption in large amounts can damage the heart and such cardiac injury can progress to severe heart failure (Gardner and Mouton, 2015). Taken together, exosomal Hsp60, derived from cardiomyocytes, more or less, plays a detrimental role in cardiac injuries associated with chronic alcohol consumption. Consequently, circulating exosomal Hsp60 may be utilized as a biomarker to reflect the extent of alcohol-induced injury in cardiac cells and to predict prognosis of regular alcohol drinkers.

#### Hsp72

Both *in vitro* and *in vivo* studies showed that the JAK/STAT3 pathway is important in preventing cardiac fibrosis and remodeling (Wang et al., 2002; Harada et al., 2005). However, expression and activation of STAT3 were severely decreased in failing hearts, partially aggravating heart failure (Podewski et al., 2003). A recent study demonstrated that exosomal Hsp72 could bind to the TLR2 on the surface of target cells and then increase STAT3 phosphorylation (Chalmin et al., 2010). This suggested that fibrosis might be inhibited in failing hearts and, to a certain degree, progression of heart failure delayed, by increasing plasma levels of exosomal Hsp72. Exercise was shown in several studies to induce Hsp72 release into the circulation, although the specific mechanisms remain unknown (Walsh et al., 2001; Febbraio et al., 2002; Fehrenbach et al., 2005). A previous study in rats indicated that sympathetic nervous system activation of alpha-1 adrenergic receptors was responsible for the increased Hsp72 in circulating plasma exosomes after exposure to acute stressors. Considering that exercise can stimulate the sympathetic nervous system and activate adrenergic receptors (Laxson et al., 1989), it is reasonable, albeit without direct experimental evidence, to speculate that exercise facilitates exosomal Hsp72 release, mediated by the sympathetic nervous system. Therefore, exercise may be applied in rehabilitation of heart failure, in part, because it increases circulating exosomal Hsp72, to protect failing hearts.

### Proteins related to the renin-angiotensin system (RAS)

As all components of the RAS are generated or activated in almost all tissues, it is now considered not only an endocrine but also a paracrine or autocrine system (Kumar et al., 2012). In the progression of heart failure, RAS was activated and, in turn, RAS activation aggravated heart failure, by increasing afterload or promoting cardiac remodeling (Zhou et al., 2016). In heart failure, increased Ang II levels were shown to facilitate secretion of exosomes by fibroblasts. Further experiments showed that, by activating MAPKs and Akt, these exosomes induced neighboring cardiomyocytes to release more Ang II and express more AT_1_R and AT_2_R, leading to myocardial hypertrophy (Lyu et al., 2015). Cardiac pressure overload, resulting from diseases like hypertension or aortic valve stenosis, is a universally accepted factor leading to heart failure. A recent study demonstrated that pressure overload facilitated release of exosomes loaded with AT_1_R, mainly from cardiomyocytes. Exogenous administration of these AT_1_R-enriched exosomes can target resistance vessels and cardiomyocytes. As a consequence, under conditions of increased plasma Ang II or pressure overload induced by hypertension, elevation of circulating AT_1_R-loaded exosomes may aggravate cardiac dysfunction during blood pressure overload (Pironti et al., 2015). Hence, in addition to commonly used drugs, such as angiotensin-converting enzyme inhibitors or angiotensin receptor blockers, strategies aiming to prevent release of detrimental exosomes might attenuate progression of cardiac dysfunction caused by RAS activation.

### Chemokine receptor type 4 (CXCR4)

CXCR4, an alpha-chemokine receptor specific for stromal-derived-factor-1, was reported to prevent heart failure by alleviating ventricular remodeling and increasing capillary density (Larocca et al., 2012). Overexpression of CXCR4 in MSCs can induce MSCs to release exosomes (isolated by precipitation) enriched in CXCR4 (Exo^CR4^). *In vitro* experiments showed that Exo^CR4^ inhibited hypoxia-induced apoptosis of cardiomyocytes via increased p-Akt levels and decreased caspase 3 activation. In addition, Exo^CR4^ can facilitate tube formation by enhancing the generation of vascular endothelial growth factor. Implantation of MSC sheets pretreated with Exo^CR4^ were shown to improve cardiac function by promoting angiogenesis, reducing infarct size and inhibiting cardiac remodeling after MI (Kang et al., 2015). MSC patches pretreated with both Exo^CR4^ and Exo^CR4^ are therefore promising strategies for treating ischemic heart disease.

### Tumor necrosis factor α (TNF-α)

Loss of cardiomyocytes during acute AMI accelerates ventricular remodeling and, finally, leads to heart failure. It was shown that TNF-α released early in the course of AMI aggravated myocardial injury and cardiac dysfunction (Li et al., 1999). Under hypoxia, cardiomyocytes were induced by hypoxia inducible factor 1α to generate more TNF-α, which was encapsulated by exosomes and then released. Incubating these TNF-α containing vesicles with cardiomyocytes increased apoptosis in cardiomyocytes (Yu et al., 2012). Thus, preventing release of this exosomal TNF-α may help to mitigate myocardial injuries and improve cardiac dysfunction after AMI.

### Sonic hedgehog (Shh)

Bone marrow-derived CD34+ stem cells were reported to have therapeutically beneficial effects in AMI (Quyyumi et al., 2011). The therapeutic effects of CD34+ cells were attributed to facilitating angiogenesis and vasculogenesis in ischemic regions of cardiac muscle (Jujo et al., 2008). The major advantage of utilizing CD34+ cells in patients is that they have little toxicity and seldom induce inflammation, as they are autologous. However, it is difficult to achieve the full therapeutic potential of CD34+ cells in the elderly and chronic smokers, because circulating CD34+ cell levels are negatively affected by factors like smoking and age (Vasa et al., 2001; Kondo et al., 2004). Therefore, efforts should be made to maintain the potency of autologous CD34+ cells in elderly patients or chronic smokers suffering from AMI. The Shh protein is a well-recognized angiogenic factor that was shown to mitigate ischemia-induced tissue damage and cell death. Shh-modified human CD34+ cells (CD34^Shh^) improved cardiac function after AMI by increasing capillary density and decreasing infarct size, compared with unmodified CD34+ cells. The underlying mechanism was that, after being injected, there was increased cardiac retention of CD34^Shh^, followed by production of a large amount of Shh-rich exosomes (Mackie et al., 2012). This suggested that exogenous administration of Shh-rich exosomes, prepared in advance, could potentially facilitate improvement of cardiac function after AMI.

### Heat shock factor 1 (HSF-1)

In AMI patients, stem cell transplantation is an additional way to compensate for cardiomyocyte loss induced by ischemia and reperfusion therapy. Because most stem cells are undetectable in the heart several hours after transplantation (Wu et al., 2011), the therapeutic effects of stem cells might be correlated with their paracrine factors, such as exosomes. It was shown that transplantation of Sca-1+ stem cells pretreated with a heat shock (^HS^Sca-1+ cells) in the ischemic myocardium decreased cardiomyocyte apoptosis and alleviated cardiac fibrosis, thereby improving global cardiac functions. Further experiments revealed that cardiomyocytes internalized exosomal HSF-1 (isolated by precipitation) from the ^HS^Sca-1+ stem cells and thus had increased levels of Hsp70, a chaperone-mediating cytoprotective protein. These effects were related to modulation of miRNA-34a, by trimethylation of histone H3 Lys27 on its promoter. HSF-1 inhibited expression of miRNA-34a, which can decrease Hsp70 levels via post-translational regulation (Feng et al., 2014a).

### Clusterin

After AMI, the pericardial fluid can induce vascular growth and cardiac tissue regeneration (Limana et al., 2010) via the epithelial to mesenchymal transition (EMT) (Zhou et al., 2011; van Wijk et al., 2012). Clusterin is a heterodimeric glycoprotein expressed ubiquitously in human tissues. Intravenous clusterin administration can decrease infarct size (Van Dijk et al., 2010) perhaps by inducing EMT (Lenferink et al., 2010) under conditions of MI. A recent study detected exosomes in pericardial fluids from non-infarcted patients (PFC) and patients with acute MI (PFMI). However, clusterin was found in exosomes isolated from patients with PFMI, but not PFC. This study also showed that, by promoting epicardial cells to undertake EMT, addition of clusterin to the pericardial sac increased arteriolar length and density and decreased apoptosis in the peri-infarct area, thus improving cardiac function. Therefore, it is very likely that exosomal clusterin, released during PFMI, exerts cardioprotective and regenerative effects in the infarcted heart (Foglio et al., 2015).

## Other types of exosomal cargoes

### Exosomal lipids

Lipid sorting results in enrichment of some phospholipids, such as sphingomyelin, phosphatidylserine, phosphatidylinositol and phosphatidic acids, and neutral lipids, such as free cholesterol and ceramides, in exosomes, compared with in the parental cells (Record et al., 2014). Exosome internalization in cells led to concentration of bioactive lipids into MVBs of the recipient cells (Subra et al., 2010). Exosomal 15d-PGJ2 can accumulate in the MVBs to reach approximate concentrations as high as 50 μM. As prior studies showed that 15d-PGJ2 at 1–10 μM was sufficient to activate PPAR-γ (Zhang et al., 2009; Wu et al., 2016), 15d-PGJ2 derived from exosomes should be sufficient to activate PPAR-γ-related pathways, after being released by MVBs in recipient cells. Moreover, it was suggested that exosomal 15d-PGJ2 was already activated by PPAR-γ before entering recipient cells, because PPAR-γ was also enriched in exosomes from human plasma (Looze et al., 2009). Because PPARγ activation was demonstrated to attenuate cardiomyocyte hypertrophy induced by high glucose and insulin (Chen et al., 2015; Peng et al., 2015), exosomal 15d-PGJ2 is a promising strategy for preventing cardiomyopathy in patients with diabetes.

### Exosomal long non-coding RNAs (lncRNAs)

lncRNAs are a recently discovered type of non-coding RNA that, unlike miRNAs, are >200 nucleotides long and poorly conserved. Their functions are largely uncharacterized in humans and other vertebrate animals (Bhartiya and Scaria, 2016). lncRNAs are involved in regulating homeostasis and pathophysiological processes by acting on neighboring protein-coding genes (Uchida and Dimmeler, 2015). A recent study demonstrated that exosomes were highly enriched in lncRNAs (Batagov et al., 2011; Kogure et al., 2013). Using microarray analysis and quantitative PCR to analyze plasma from CAD patients, lncRNA AC100865.1 (signal intensity >8, fold change >2.5, *P* < 0.005, compared with in normal subjects) was identified as a novel biomarker for predicting CAD. Packaged in extracellular vesicles, lncRNA AC100865.1 is relatively stable, even when exposed to room temperature or freeze–thaw cycles. Using a diagnostic model with Fisher's criteria, taking risk factors into account, the optimal sensitivity of lncRNA AC100865.1 for CAD was 78.05% and the specificity was 86.49% (Yang et al., 2015).

## Conclusions and future perspectives

It has become increasingly apparent that exosomes and their contents, discharged by various cells, are crucial in local and remote intercellular crosstalk. In the failing heart, diverse exosomal miRNAs, proteins and lipids contribute to cardiomyocyte hypertrophy, impaired angiogenesis and ECM degradation. Thus, they are very promising markers for predicting MI or other CVDs at different stages, although present techniques for their detection are relatively complicated and time-consuming. We believe that applying advanced technologies to exosome isolation and content examination will soon lead to development of improved methods. Already, microfluidic-based techniques are effective for exosome isolation (Li et al., 2017) and simple, rapid and sensitive miRNA detection (Arata et al., 2014) and could be applicable to point-of-care testing. In addition to the widely studied miRNAs, other RNAs (lncRNAs or circular RNAs) encapsulated in exosomes also participate in regulation of homeostasis and pathophysiological processes. Their roles in CVDs remain mostly unclear and must be further addressed in future research. In addition, results in animals and humans are not always consistent among research groups. For example, exosomal miRNA-146a derived from ECs (Halkein et al., 2013) and that from CDCs (Ibrahim et al., 2014) had completely opposite effects on the microvasculature in PPCM and MI, respectively. Also, reported alterations in circulating miRNAs were inconsistent in similar studies examining MI patients (Corsten et al., 2010; D'Alessandra et al., 2010; Kuwabara et al., 2011). Taken these inconsistencies into consideration, further experiments should focus not only on exosome-induced cell–cell communication under different pathological status but also on resolving inconsistent findings, to ultimately achieve translation from bench to bedside.

As the primary effective components, stem cell-derived exosomes and exosomes extracted from cells pretreated with stressors appear to have regenerative properties when applied to cardiomyocytes, ECs or failing hearts. Compared with directly administered stem cells, exosomes have potentially superior properties, such as immunogenicity, safety, and efficacy, and they also are associated with fewer ethical concerns. In addition, it is possible to obtain a large amount of exosomes by calcium stimulation (Savina et al., 2003) or artificial synthesis (Radenkovic et al., 2016), providing adequate material for clinical use. Hence, exosomes may be used as drug delivery systems for carrying efficient and specific cargoes selectively to locations or cell types within the diseased heart. Future clinical studies are needed to confirm the therapeutic promise of exosome-based treatments in CVDs.

However, there are also several limitations in this field. First, current techniques for exosome isolation are unable to guarantee high yields, efficiency, purity and throughput. Second, in previous research, there was a lack of specific tools to characterize exosomes, thus limiting understanding of their functions. Therefore, we suggest that exosome preparations, which are co-enriched in CD63, CD81, CD9, and endosome markers (syntenin-1, TSG101), be identified according to the definition described by Kowal et al. before they are used for functional testing (Kowal et al., 2016). Finally, after intravenous injection, exosomes accumulate primarily in the liver, spleen, gastrointestinal tract and lung, while smaller portions of the exosomes remain in the heart (Wiklander et al., 2015). Further efforts should be focused on overcoming these limitations.

## Author contributions

Substantial contribution to the concept and interpretation of available evidence (all authors); drafted the manuscript (JX and GC); critically revised the manuscript for important intellectual content (JX and GC); gave final approval of the manuscript for publication (all authors). All authors agree to be accountable for all aspects of the work and for ensuring that questions related to the accuracy or integrity of any part of the work are appropriately investigated and resolved.

### Conflict of interest statement

The authors declare that the research was conducted in the absence of any commercial or financial relationships that could be construed as a potential conflict of interest.
