# Erratum: Exosomes: A Rising Star in Failing Hearts

**DOI:** 10.3389/fphys.2017.00620

**Published:** 2017-08-22

**Authors:** Frontiers Production Office

**Affiliations:** Frontiers Media SA Lausanne, Switzerland

**Keywords:** exosomes, cardiac dysfunction, microRNAs, proteins, heat shock proteins, stem cells

Due to a production error the word “Failing” in the title of the article was accidentally published as “Falling.” The publisher apologizes for this mistake. The original version of this article has been updated.

